# Counseling for Prenatal Congenital Heart Disease—Recommendations Based on Empirical Assessment of Counseling Success

**DOI:** 10.3389/fped.2020.00026

**Published:** 2020-02-26

**Authors:** Alexander Kovacevic, Andreas Simmelbauer, Sebastian Starystach, Michael Elsässer, Andreas Müller, Stefan Bär, Matthias Gorenflo

**Affiliations:** ^1^Department of Pediatric and Congenital Cardiology, Heidelberg University, Hospital, Heidelberg, Germany; ^2^Max Weber Institute for Sociology, Ruprecht Karls University Heidelberg, Heidelberg, Germany; ^3^Department of Gynecology and Obstetrics, Heidelberg University Hospital, Heidelberg, Germany

**Keywords:** fetal cardiology, Congenital heart disease (CHD), counseling, empirical assessment, parental needs

## Abstract

**Objectives:** Empirical assessment of parental needs and affecting factors for counseling success after prenatal diagnosis of congenital heart disease (CHD).

**Methods:**Counseling success after fetal diagnosis of CHD was assessed by a validated standardized questionnaire. The dependent variable “Effective Counseling” was measured in five created analytical dimensions (1. “Transfer of Medical Knowledge—*ToMK*”; 2. “Trust in Medical Staff—*TiMS*”; 3. “Transparency Regarding the Treatment Process—*TrtTP*”; 4. “Coping Resources—*CR*”; 5. “Perceived Situational Control–*PSC*”). Analyses were conducted with regard to influencing factors and correlations.

**Results:** Sixty-one individuals (*n* = 40 females, *n* = 21 males) were interviewed in a tertiary medical care center. Median gestational age at first parental counseling was 28 + 6 weeks. Parental counseling was performed four times (median), mostly by pediatric cardiologists (83.6%). Overall counseling was successful in 46.3%, satisfying in 51.9%, and unsuccessful in 1.9%. Analyses of the analytical dimensions show that counseling was less successful for TOMK (38.3%) and PSC (39%); success rates were higher if additional written information or links to web sources were provided (60 and 70%, respectively). Length of consultation was positively correlated to counseling success for ToMK (*r* = 0.458), TrtTP (*r* = 0.636), PSC (*r* = 0.341), and TiMS (*r* = 0.501). Interruptions were negatively correlated to the dimensions TiMS (*r* = −0.263), and TrtTP (*r* = −0.210). In the presence of high-risk CHD (37.5%) overall counseling success was lower (26.1%). By cross table analysis and to a low degree of positive correlation in one dimension (ToMK; *r* = 0.202), counseling tends to be less successful for ToMK, TrtTP, and TiMS if parents have not been counseled by cardiologists. Analyses regarding premises show a parental need for a separate counseling room, which significantly impacts ToMK (*r* = −0,390) and overall counseling success (*r* = −0.333). A language barrier was associated with lower success rates for ToMK, TiMS, and CR (21.4, 42.9, and 30.8%).

**Conclusions:** Data from this multidisciplinary study indicate that parents after fetal diagnosis of CHD need uninterrupted counseling of adequate duration and quality in a separate counseling room. Providing additional written information or links to adequate web sources after initial counseling seems necessary. High-risk CHD needs more attention for counseling. There is a trend towards more counseling success if provided by cardiologists.

## Introduction

Congenital heart disease (CHD) affects nine per 1,000 live births and represents the most common congenital anomaly in newborns (1). Antenatal detection of CHD by pediatric or fetal cardiologists, maternal fetal medicine (MFM) specialists, and sonographers may improve survival postnatally (2–5).

Besides an accurate diagnosis of the malformation by fetal echocardiography, effective parental counseling is crucial, thus outlining available treatment options, providing a clear picture of the prognosis, and finally helping the parents to reach decisions, which are best for them—all ideally shortly after suspecting CHD in the fetus (6).

However, in view of this complexity, relatively little research has focused specifically on providing the most effective counseling techniques (7). Published guidelines do not seem to be fully sufficient, and more parental focused counseling seems to be required (7–9).

Therefore, one objective of this study was to gather new insights about the multiple dimensions of parental counseling after prenatal diagnosis of CHD. For this analysis, we developed a new research tool in a multidisciplinary setting. We aimed to explore parental needs and affecting factors for counseling success, with the intention to suggest recommendations based on this empirical assessment.

## Methods

### Design and Analysis

A questionnaire was developed by multidisciplinary cooperation (pediatric and fetal cardiologists, MFM specialists, and sociologists). Methodological aspects on how we created, pretested, and validated this research tool were described by our group earlier (10) (see also Supplementary File 1: statistical aspects).

We measured the dependent variable “Effective Counseling” in five created analytical dimensions [1. “Transfer of Medical Knowledge” (TiMK); 2. “Trust in Medical Staff” (TiMS); 3. “Transparency Regarding the Treatment Process” (TrtTP); 4. “Coping Resources” (CR); 5. “Perceived Situational Control” (PSC)]. Each dimension with corresponding item-sets was developed based on literature search as well as analysis of daily practice experiences (6–9, 11–15). As independent variables, we constructed questions on social, spatial, temporal, and informational aspects. For overall and each dimension's success, separate sum scores were used to differentiate between the three levels of “Counseling Success”: 1. successful, 2. satisfying, and 3. unsuccessful.

In addition, all parents had the possibility to comment on the questionnaire and to make open comments, both in free-text fields.

Data analyses were conducted with regard to influencing factors and correlations using IBM SPSS® V. 25. Frequency analysis, correlation, and cross table calculations as well as calculations of average, standard deviation, and median were performed.

### Setting and Study Population

The study was conducted at a tertiary care medical center involving the Departments of Pediatric Cardiology/Congenital Heart Disease, Obstetrics and Gynecology as well as the Max-Weber Institute for Sociology.

Ethical approval was obtained by the local ethics committee of the Medical Faculty (date: 17.06.2016; reference number: S-250/2016). All participants gave their written informed consent.

The inclusion criteria were parental age at least 18 years (and custody of the child), CHD detected during antenatal screening (2009–2016), chromosomal abnormalities included if not associated with unfavorable outcome as sole diagnosis (such as Trisomy 13 or 18). Fetuses with extracardiac abnormalities influencing overall postnatal outcome were excluded.

Further details were published by our group previously (10).

## Results

Sixty-one individuals (*n* = 40 female, *n* = 21 male) were interviewed. Gestational age at diagnosis and first parental counseling was 28 + 6 weeks in median. Parental counseling was performed overall four times in median (range 1–17), mainly by pediatric/fetal cardiologists (83.6%) and/or MFM specialists (by *n* = 2 pediatric/fetal cardiologists, *n* = 3 MFM specialists). Parents of fetuses with chromosomal abnormalities were uniformly offered a separate counseling by a geneticist, two accepted this (in our country cardiologists and MFM specialists may counsel parents on genetic syndromes if related to their field and if qualified by certification of their medical association).

Table 1 summarizes the fetal cardiac diagnoses and chromosomal abnormalities, if present. Of the cases, 37.5% were high risk, 42.5% were moderate risk, and 20% were low risk CHD.

**Table 1:** Overview of cardiac diagnoses, associated chromosomal abnormalities, and grading of the heart defects [according to Allan and Huggon (6)].

**Fetal cardiac diagnosis**	**Grading of severity**	**Chromosomal abnormality Y/N**	
ASD	1	Y	Trisomy 21
Hydrops, tricuspid regurgitation	1	N	
Suspicion for coarctation, ventricular disproportion	1	N	
VSD	1	N	
VSD	1	N	
VSD	1	N	
VSD	1	Y	^*^Reciprocal translocation chromosome 1 and 7; deletions: 1q43 and 7p15.3 - p21.1
VSD	1	Y	^*^Cystic fibrosis
Coarctation	2	N	
AVSD	2	Y	Trisomy 21
AVSD	2	N	
AVSD	2	N	
AVSD	2	Y	Trisomy 21
AVSD	2	N	
AVSD	2	Y	Trisomy 21
AVSD	2	N	
AVSD	2	Y	Trisomy 21
Coarctation	2	N	
DORV	2	N	
DORV	2	N	
TOF	2	Y	Trisomy 21
Hypoplastic aortic arch, coarctation	2	N	
TOF	2	N	
TOF, right aortic arch	2	Y	DiGeorge-syndrome
Tricuspid atresia Ib	2	N	
Coarctation	3	Y	Turner syndrome
Coarctation	3	N	
Complex TGA	3	N	
HLHS	3	N	
Hypoplastic aortic arch, coarctation	3	N	
Hypoplastic aortic arch, borderline LV	3	N	
Non compaction cardiomyopathy	3	N	
PA, IVS	3	N	
PA, VSD	3	N	
PA, VSD	3	N	
PA, VSD, TGA	3	N	
PA, VSD, MAPCAs	3	N	
PA, IVS, sinusoids	3	N	
TGA	3	N	
IAA, VSD	3	N	

*Note. 1 = low risk CHD = little or no effect on life or lifespan (n = 8)*.

*2 = moderate risk CHD = low mortality for surgery, but likely to affect long-term survival (n = 17)*.

*3 = high risk CHD = a high mortality for surgery or repeated surgeries likely during childhood or likely to be compromised cardiologically as young adults (n = 15)*.

*HLHS, hypoplastic left heart syndrome; TGA, transposition of the great arteries; TOF, tetralogy of Fallot; VSD, ventricular septal defect; PA, pulmonary atresia; IVS, intact ventricular septum; MAPCAs, main aortopulmonary collateral arteries; AVSD, atrioventricular septal defect; IAA, interrupted aortic arch*.

* *Postnatal diagnosis*.

Most parents (just under 80%) felt that the time between the suspected diagnosis and the consultation with a specialist was short. Of the parents, 73% found the duration of the medical consultation to be appropriate. In 18% of the cases, the counseling was frequently interrupted. Of the parents, 37.5% received written information.

Using the sum score to assess counseling success, overall counseling was successful in 46.3% and satisfying in 51.9%; 1.9% of the parents were dissatisfied.

Further analyses of the five created analytical dimensions show that “Transfer of Medical Knowledge” (38.3% of parents perceived this as successful, 60.0% as satisfying, and 1.7% as unsuccessful) and “Perceived Situational Control” (39.0% of parents perceived this as successful, 39.0% as satisfying, and 22.0% as unsuccessful) are considered less successful (for “Transfer of Medical Knowledge overall adequate) compared to the remainder (for “Trust in Medical Staff,” “Transparency Regarding the Treatment Process,” and “Coping Resources” counseling was perceived more effective with success rates of 69.5, 60.0, and 56.9%) (Table 2).

**Table 2:** Counseling success in percent of total (a) “Overall Counseling Success” and (b) “Counseling Success” in the five analytical dimensions (*n* = 61).

	**Counseling success**		
	**Successful**	**Satisfying**	**Unsuccessful**
(a) Overall counseling success	46.3%	51.9%	1.9%
(b) Dimensions			
Transfer of medical knowledge	38.3%	60.0%	1.7%
Trust in medical staff	69.5%	27.1%	3.4%
Transparency regarding the treatment process	60.0%	36.7%	3.3%
Coping resources	56.9%	37.9%	5.2%
Perceived situational control	39.0%	39.0%	22.0%

Counseling success rates were higher for the dimensions “Transfer of Medical Knowledge” and “Perceived Situational Control” (60.0 and 70.0%, respectively; *n* = 10), if after initial counseling additional written information or links to adequate web sources explaining the nature, therapy, and outcomes of the heart defect were provided.

**Figure 1:**
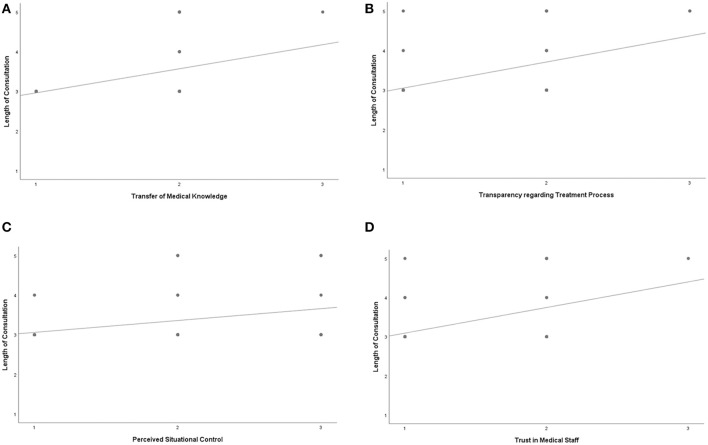
(A–D)*Length of consultation was positively correlated to **(A)** Transfer of Medical Knowledge *r* = 0.458, *p* = 0.000, **(B)** Transparency regarding treatment process *r* = 0.636, *p* = 0.000, **(C)** Perceived Situational Control *r* = 0.341, *p* = 0.012, **(D)** Trust in Medical Staff *r* = 0.501, *p* = 0.000 x-axis: 1 = successful to 3 = minor success y-axis: 1 = appropriate to 5 = too short.

Length of consultation was positively correlated to counseling success for “Transfer of Medical Knowledge” (*r* = 0.458), “Transparency Regarding the Treatment Process” (*r* = 0.636), “Perceived Situational Control” (*r* = 0.341), and “Trust in Medical Staff” (*r* = 0.501) (Figures 1A–D).

However, the absolute number of parental consultations had no effect on counseling success in any dimension.

**Figure 2:**
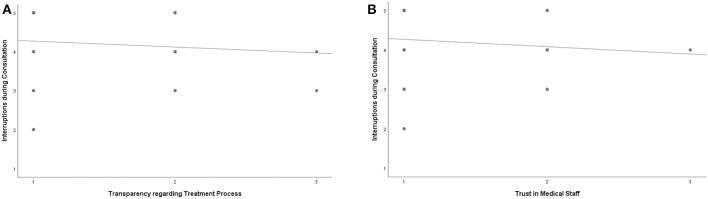
**(A,B)** *Interruptions during consultations were negatively correlated to **(A)** Transparency Regarding Treatment Process *r* = −0.210, *p* = 0.110 **(B)** Trust in Medical Staff *r* = −0.263, *p* = 0.044 x-axis: 1 = successful to 3 = minor success y-axis: 1 = highly occurred to 5 = did not occur. *Contrary to the common point clouds in scatter plots for variables with many expressions, the figures show the occurring combinations of the two variables with five and three characteristics, respectively (respectively, the slope line); consequently, one point stands for the responses of several participants.

Interruptions during consultations were negatively correlated to the dimensions “Trust in Medical Staff” (*r* = −0.263) and “Transparency Regarding the Treatment Process” (*r* = −0.210) (Figures 2A,B).

If cardiac diagnosis was graded as high-risk CHD, overall counseling success was lower at 26.1% (Table 3).

**Table 3:** Overall counseling success in percent of total depending on the grading of the diagnosed congenital heart disease (CHD) (*n* = 61).

	**CHD grading**		
	**Low-risk CHD**	**Moderate risk-CHD**	**High-risk CHD**
Counseling:			
Successful	80.0%	52.4%	26.1%
Satisfying	20.0%	42.9%	73.9%
Unsuccesful	-	4.8%	-

If parental native language was not German, counseling success rates were lower for overall success and in all dimensions (Table 4); (in these cases, counseling was performed in German as parental language skills were obviously good enough, and the parents' understanding seemed adequate during counseling sessions; no parental questionnaires were included if a translator was necessary, as we would not have had certainty about the translation quality).

**Table 4:** Effect of parental native language on overall counseling success and on the five analytical dimensions.

	**Native language German**	**Native language other than German (n=14)**
	Success rate:	
(a) Overall counseling success	53.7%	23.1%
(b) Counseling success in dimensions	Success rate:	
Transfer of medical knowledge	43.5%	21.4%
Trust in medical staff	77.8%	42.9%
Transparency regarding treatment process	65.2%	42.9%
Coping resources	64.4%	30.8%
Perceived situational control	42.2%	28.6%

Answers on questions regarding premises clearly showed a parental need for a separate room for counseling, which significantly impacts “Transfer of Medical Knowledge” (*r* = −0.390, *p* = 0.002) and overall counseling success (*r* = −0.333, *p* = 0.015).

By cross table analysis and to a low degree of positive correlation in one dimension (“Transfer of Medical Knowledge”; *r* = 0.202), counseling tends to be less successful for the three dimensions: “Transfer of Medical Knowledge,” “Transparency Regarding the Treatment Process,” and “Trust in Medical Staff,” if parents have not been counseled by cardiologists [standardized residuals greater than or equal to 1 (in terms of amount) in cross tabulation, but in a small number of cases of counseling, not provided by the pediatric cardiologists].

For parents of children with chromosomal abnormalities (*n* = 15 parents) (see Table 1 for details of genetic findings), the overall success of counseling was higher (*r* = −0.223), as is the specific success in the dimensions “Trust in Medical Staff” (*r* = −0.201) and “Transparency Regarding Treatment Process” (*r* = −0.292).

Preliminary analyses of the open questions show that parents evaluated the questionnaire very positively, in general. The perception was a strong involvement in the process of decision making. The parents noted that counseling was not only for informational purposes but also helped to cope with the situation after fetal diagnosis of CHD.

## Discussion

Antenatal detection of CHD may improve postnatal survival in newborns with critical heart disease, in particular, in duct-dependent lesions (2–5). Prenatal detection rates have been increasing due to technical advances, evolution of fetal echocardiography, and consecutive special training of sonographers, cardiologists, and MFM specialists (16).

Different associations and societies have published recommendations for parental counseling after fetal diagnosis of CHD [American Heart Association (AHA), Association for European Pediatric and Congenital Cardiology (AEPC), International Society of Ultrasound in Obstetrics and Gynecology (ISUOG)] (7, 8, 17, 18). Besides an accurate cardiac diagnosis, a clear and truthful picture of the prognosis must be provided, outlining available postnatal, and in selected fetuses, prenatal treatment options, thus finally helping the parents to reach decisions that are best for them—all this ideally shortly after suspecting CHD in the fetus (7, 19–21). The option of termination of pregnancy must be discussed according to the country's legislation.

Further potential diagnostic limitations and uncertainties (affected by gestational age, fetal position, and maternal habitus) should be discussed as well as extra-cardiac anomalies and the potential effect on postnatal management and prognosis. Also, independent risk factors such as growth restriction and prematurity need to be discussed. Finally, delivery planning and expected postnatal management (need for prostaglandins, diagnostics, cardiac catheterization, and cardiac surgery) need to be outlined.

Regarding the objective quality of counseling, it cannot generally be expected that consultants uniformly will have excellent communicative skills or skills in the assessment of the parents' body language during counseling to be perceptive in assessing how the information is received in order to be able to adapt it during the counseling session (7).

Most professionals advocate a non-directive counseling. However, it can be assumed that during many counseling sessions, selective information is provided, sometimes imposing personal bias into the discussion (11).

Furthermore, published guidelines for parental counseling after fetal diagnosis of CHD do not seem to be fully sufficient in terms of satisfying parental needs. Arya et al. could demonstrate that parental needs for counseling for fetal CHD often differ from the information cardiologists provide. Significant differences were found, e.g., for topics regarding the child's quality of life, future number of lifetime surgeries, or if the child will have exercise limitations later in life (9). Menahem et al., in their study, describe similar results concluding that parents seemed more satisfied if the child's functional outcome, rather than the anatomical detail of the cardiac diagnosis, is in the focus during counseling sessions (12). This finding is supported by Carlsson et al. in their study, parents described the information given by cardiologists as often too overwhelming and complex. The child's quality of life was an important topic for the parents. The Internet was considered, in general, as low-quality information source. Providing written information was an important parental need after repeated sessions (13). Bratt et al. emphasize that high-quality web-based information in addition to written information is valued by parents. They also found that a short waiting time from the first suspicion of fetal CHD to the final cardiac diagnosis was important for parents and that counseling was better perceived if it was undertaken in the parental native language (14).

In view of this evident complexity, relatively little research has focused specifically on providing the most effective counseling techniques (7). This highlights the need for predefined guidelines and standardization among tertiary centers.

Therefore, the objective of our study was to explore parental needs and affecting factors for counseling success after prenatal diagnosis of congenital heart disease. Based on our analyses, we aimed to propose guidelines based on this empirical assessment.

A multidisciplinary cooperation between sociologists, cardiologists, and MFM specialists resulted in the development and consecutive validation of a questionnaire (10). We believe that this interdisciplinary approach significantly adds to the strength of this study. Similar multidisciplinary approaches are shown to be extremely helpful and may contribute to diagnostic and therapeutic improvement in different medical fields (22).

With this newly developed research tool, we can assess counseling success in different dimensions: “Transfer of Medical Knowledge,” “Trust in Medical Staff,” “Transparency Regarding the Treatment Process,” “Coping Resources,” and “Perceived Situational Control.”

In the present study, 46.3% of the parents assessed counseling as successful. “Trust in Medical Staff” was high with 69.5%, and 51.9% felt that counseling was at least satisfying. “Transfer of Medical Knowledge” was shown to be overall adequate (satisfying = 60%; successful = 38.3%). However, parental “Perceived Situational Control” often seems impaired indicating a parental need for further and continuous support throughout the pregnancy. Early inclusion of cardiac specialist nurses, psychologists, or social workers seems necessary in selected cases. For the remaining dimensions (“Transparency Regarding the Treatment Process,” “Coping Resources”), parents felt that counseling was more successful.

Our results show that the length of consultations should be adequate—depending on the complexity of the diagnosed CHD—as the length of consultation is positively correlated to counseling success for “Transparency Regarding the Treatment Process,” “Perceived Situational Control,” and “Transfer of Medical Knowledge.” Interestingly, the absolute number of consultations had no effect on counseling success in any dimension indicating that quality of counseling is essential. However, in another study, parents reported that oral information needs to be repeated several times (14).

We could show that interruptions during counseling sessions were negatively correlated to counseling success for “Trust in Medical Staff” and “Transparency Regarding the Treatment process”; this describes the particular sensitive and vulnerable situation parents are in during the first counseling sessions for a heart defect of their unborn child. For practical reasons, consultants should be given the requirements for continuous counseling without interruptions by medical staff.

In complex CHD, in our series, in 37.5% of the cases, overall counseling success was lower showing that these cases need more attention.

By cross table analysis and to a low degree of positive correlation in one dimension (“Transfer of Medical Knowledge”), we have a first impression that if parents are not counseled by pediatric cardiologists, counseling in three dimensions (“Transfer of Medical Knowledge,” “Transparency Regarding the Treatment Process,” and “Trust in Medical Staff”) tends to be less successful.

Further, a language barrier plays an important role. If parental native language was different from the language counseling was conducted in, success rates were lower, in particular, for “Transfer of Medical Knowledge,” for “Trust in Medical Staff,” “Coping Resources.” The presence of any kind of language barrier seems to have a significant impact on counseling success, even if unapparent as in our cohort (no parents were included after counseling with a translator, as we would not have had certainty about the translation quality). Similar findings were shown earlier, but not in this specific way for predefined dimensions with a validated research tool (14).

If a separate counseling room was not available, “Transfer of Medical Knowledge” was difficult. This finding supports the AEPC guidelines to move parents into a separate counseling room (8).

Success rates were higher for “Transfer of Medical Knowledge” and “Perceived Situational Control” if, after initial counseling, further additional written information or links to adequate web sources were provided. This supports the AHA guidelines (7) and is further supported by Bratt et al. (13) and Carlsson et al. (14). The Internet as a secondary information source is commonly used by parents, which highlights the need to provide web sources of high quality, ideally in different languages (14, 23).

## Limitations

Owing to the limited numbers, study design, and sampling method, the generalizability of our findings may be impaired. We aim to extend this study by performing a national multicenter approach to be able to draw more general conclusions by increasing numbers and thus statistical power. Further, a randomized controlled study to test our main results, which our group will initiate after finishing off the multicenter study, should be helpful. Therefore, e.g., we are aiming to launch our own Internet-based information platform on CHD in different languages. Furthermore, we will analyze parental sociodemographic and psychological factors and look for potential correlations to counseling success via the five analytical dimensions.

Parents have not been involved in the development of the questionnaire. However, there was the possibility for all participants to make comments (open questions: comments on the questionnaire, in general, and open comments).

We did not include individual skills or training of professionals as variable because we aimed to find out potential differences in counseling success depending on the physician's specialty, not between the professionals, in general.

## Conclusions

These data indicate that parents, after fetal diagnosis of CHD, need uninterrupted counseling of adequate duration and quality in a separate counseling room.

Providing written information or links to adequate web sources seem necessary, ideally in the parental native language.

There is a trend towards more counseling success if provided by cardiologists.

We found that high-risk CHD needs more attention for counseling pointing in the same direction.

Parental “Perceived Situational Control” is often impaired, highlighting the need for continuous support throughout the pregnancy, e.g., by psychologists or social workers.

It is very likely that both individual skills and different training of professionals may have a significant impact on counseling, which we did not include as variables in this study. However, our results may serve as a paradigm to improve training and, thus, service in this setting.

Further analyses of parental sociodemographic factors and psychological aspects seem worthy in order to identify additional factors affecting counseling after fetal diagnosis of CHD.

The mid-term goal is to propose evidence-based national guidelines.

## Data Availability Statement

The datasets generated for this study are available on request to the corresponding author (depending on data protection guidelines of the local Ethics Committee).

## Ethics Statement

The studies involving human participants were reviewed and approved by Ethics Committee of the Medical Faculty, Heidelberg University (date: 17.06.2016; reference number: S-250/2016). The patients/participants provided their written informed consent to participate in this study.

## Author Contributions

AK: leader, study design, data acquisition and analysis, parental counseling, and manuscript writing and review. AS: design of the questionnaire, data acquisition, and manuscript writing. SS: design of the questionnaire, methodological part, statistical analysis, and manuscript writing and review. ME and AM: design of the questionnaire, parental counseling, and careful manuscript review. SB: design of the questionnaire, methodological part, statistical analysis, and manuscript writing and review. MG: study design and careful manuscript review.

### Conflict of Interest

The authors declare that the research was conducted in the absence of any commercial or financial relationships that could be construed as a potential conflict of interest.
