# Supplementary material for: Counseling for Prenatal Congenital Heart Disease—Recommendations Based on Empirical Assessment of Counseling Success
Source: Front Pediatr. 2020 Feb 26;8:26. doi: 10.3389/fped.2020.00026 (PMC7054339; doi:10.3389/fped.2020.00026)
Supplement: Supplementary file 1 [file Data_Sheet_1.pdf]

Supplementary Material

Statistical aspects

The variable *overall counselling success* was constructed by building a sum score of the 16 items from the Likert scale and transforming it into an ordinal scaled variable defining the respective range 16-32 as successful, 33-63 as satisfying and 64-80 as unsuccessful.

Items were constructed as indicators showing success when answered as positive in the two assenting values of the Likert response format (1-5: strongly agree, partially agree, disagree, partially disagree and strongly disagree; for statistical analysis each point scale is converted into a number from one to five). In the same way, we constructed the variables for the *five dimensions of success*, whereby the number of items varied (except the dimension “perceived situational control”, that was measured by only one item).

Our decision to use an ordinal scale for success was drawn by practical considerations mainly to reduce complexity.

<u>Sum scores:</u>

Overall success= V2.2 + V2.7 + V2.10 + V2.16 + V2.17+ V2.11 + V2.12 + V2.27+ V2.13 + V2.14 + V2.15 + V2.18+ V2.23 + V2.21 + V2.20+V2.22 (α=0.920)

SumToMK=V2.2 + V2.7 + V2.10 + V2.16 + V2.17 (α=0.752)

SumTiMS=V2.11 + V2.12 + V2.27 (α=0.874)

SumTrtTP=V2.13 + V2.14 + V2.15 + V2.18 (α=0.826)

SumCR=V2.23 + V2.21 + V2.20 (α=0.806)

PSC=V2.22

Definition of success:

Overall success 16-32=successful, 33-63=satisfying, 64-80=unsuccessful

ToMK: 5-10=successful, 11-19=satisfying, 20-25=unsuccessful

TiMS: 3-6=successful, 7-11=satisfying, 12-15=unsuccessful

TrtTP: 4-8=successful, 9-15=satisfying, 16-20=unsuccessful

CR: 3-6=successful, 7-11=satisfying, 12-15=unsuccessful

PSC: 1-2=successful, 3=satisfying, 4-5=unsuccessful

Abbreviations:

ToMK - Transfer of Medical Knowledge

TiMS - Trust in Medical Staff

TrtTP - Transparency Regarding the Treatment Process

CR - Coping Resources

PSC - Perceived Situational Control
